# Effects of Soy Protein Containing of Isoflavones and Isoflavones Extract on Plasma Lipid Profile in Postmenopausal Women as a Potential Prevention Factor in Cardiovascular Diseases: Systematic Review and Meta-Analysis of Randomized Controlled Trials

**DOI:** 10.3390/nu13082531

**Published:** 2021-07-24

**Authors:** Agnieszka Barańska, Agata Błaszczuk, Wiesław Kanadys, Bożena Baczewska, Marian Jędrych, Ewelina Wawryk-Gawda, Małgorzata Polz-Dacewicz

**Affiliations:** 1 Department of Medical Informatics and Statistics with E-Learning Lab, Medical University of Lublin, 20-090 Lublin, Poland; marian.jedrych@umlub.pl; 2 Department of Virology with SARS Laboratory, Medical University of Lublin, 20-093 Lublin, Poland; agata.blaszczuk@umlub.pl (A.B.); malgorzata.polz.dacewicz@umlub.pl (M.P.-D.); 3 Specialistic Medical Center Czechow, 20-848 Lublin, Poland; wieslaw.kanadys@wp.pl; 4 Chair of Internal Medicine and Department of Internal Medicine in Nursing, Medical University of Lublin, 20-093 Lublin, Poland; bozena.baczewska@umlub.pl; 5 Department of Paediatric Pulmonology and Rheumatology, Medical University of Lublin, 20-093 Lublin, Poland; ewelina.wawryk-gawda@umlub.pl

**Keywords:** soy protein containing isoflavones, soy isoflavones extracts, cardiovascular diseases, postmenopausal women, lipid profile, TC, LDL-C, HDL-C, TAG

## Abstract

The aim of the report was to evaluate the impact of soy protein containing isoflavones and soy isoflavones extract on lipid profile in postmenopausal women, as compared with placebo or protein of milk, casein or isolated soy protein with or without trace isoflavone content. We used the following databases: MEDLINE (PubMed), EMBASE and the Cochrane Library. Quantitative data synthesis was performed by applying a random-effects model. Subgroup analysis and meta-regression were performed to assess the modifiers of treatment response. In total, in the analysis studies, 2305 postmenopausal women took part. Changes in the lipid profile showed statistically significant decreases of total cholesterol by −0.12 (95% CI: −0.21, −0.03) mmol/L, −4.64 (95% CI: −8.12, −1.16) mg/dL, *p* = 0.01 and increased HDL-cholesterol by 0.03 (95% CI: 0.00, 0.06) mmol/L, 1.15 (95% CI: 0.00, 1.93) mg/dL, *p* = 0.05, as well as in LDL-cholesterol −0.05 (95% CI: −0.11, 0.01) mmol/L, −1.93 (95% CI: −4.25, 0.39) mg/dL, *p* = 0.08 and triacylglycerols −0.07 (95% CI: −0.14, 0.00) mmol/L, −6.123 (95% CI: −12.25, 0.00) mg/dL, *p* = 0.06. Our results suggests that soy and its isoflavones can be effective in correction changes in lipid metabolism in postmenopausal women and may favorably influence in preventing cardiovascular events.

## 1. Introduction

Cardiovascular diseases (CVD) continue to be the number one cause of morbidity and mortality of women over 50 years of age—accounting for over one third of total deaths [1]. Before menopause, CVD is infrequent which suggests that female hormones and metabolism offer protection. After natural menopause or bilateral ovariectomy, as a result of estrogen deficiency, a progress of changes in the metabolism of women is observed. This leads to an incidence of many metabolic syndrome features, including accumulation of fat mass in the abdominal compartment, transition to a more atherogenic lipid profile, hyperinsulinemia, insulin resistance and glucose intolerance [2,3,4,5]. The consequence of these changes is an increased risk of coronary heart disease (CHD), stroke and other atherosclerotic vascular disease, including peripheral arterial disease, atherosclerotic aortic disease and carotid artery disease [6,7]. Dyslipidemia is one the most important risk factors for CVD, which can be corrected and prevented. During the menopausal transition (within the 1-year interval before and after the final menstrual period), a substantial increase of total cholesterol (TC), LDL-cholesterol (LDL-C) and apolipoprotein B has been demonstrated. This is associated with decreased circulating estrogen levels [8]. The postmenopausal situation, allied with the acceleration of several aging processes deepens further alterations in lipid profiles, among others: increase in TC, LDL-C and triacylglycerol (TAG) and reduction of HDL-cholesterol (HDL-C) [8,9,10]. Lipid disorders can accelerate the atherosclerosis process and its consequences, such as heart failure and coronary atherosclerosis. Several meta-analyses have shown that reduction of serum LDL-C by about 5–6% and a 3% increase in HDL-C are associated with improved cardiovascular outcomes [11,12].

Modification by diet and lifestyle of risk factors, particularly dyslipidemia, remains the cornerstone of therapy. Soy food and its constituent protein and isoflavones have received widespread attention for their potential role in CVD risk improvement [13,14]. In 1999, the FDA concluded that soy protein included in a diet low in saturated fat and cholesterol may reduce the risk of CHD by decreasing blood cholesterol levels [15]. Over the past two decades, many randomized controlled studies have been conducted of the effects of soy on lipids and other cardiovascular markers, but their outcomes remained inconsistent and controversial. This was reflected in the recommendations of various societies and associations of scientific standing [16,17,18,19,20]. The latest reports indicate that soy and isoflavone consumption is beneficial [21]. The results support promoting soy intake as part of a healthy diet and suggest the ability of both extracted isoflavones and soy protein with isoflavones to modulate the lipid profile and bring about benefits in preventing cardiovascular events [22].

In our systematic review and meta-analysis, we focused on the assessment of the effect of isolates of soy protein rich in isoflavones, as well as soy isoflavones extract on the lipid profile in postmenopausal women.

## 2. Materials and Methods

### 2.1. Search Strategy and Study Selection

Based on the PRISMA guidelines, we conducted searches of website electronic databases: MEDLINE (PubMed), EMBASE and the Cochrane Library up to January 2020 to identify RCTs investigating the effect of soy isoflavones on blood lipid profile [23]. The selected publications were analyzed according to the PRISMA checklist (supplementary Table S1: PRISMA 2020 Checklist). The following word search terms were used in various combinations: soy proteins, soy isoflavones, genistein, daidzin, lipids, lipoprotein, lipid profile, cholesterol, TC, HDL, LDL and TAG. Additionally, we searched the reference lists of the included studies and relevant reviews. All articles included in this work were limited to the English language. Articles were initially evaluated according to title and/or abstract. In turn, potentially appropriate works that met all the selection criteria were selected and their full texts were read in order to gather detailed information.

Studies were considered eligible for inclusion in the meta-analysis if they met all of the following criteria: double blind randomized parallel-group controlled trials; controlled against placebo or comparator treatment; the follow-up period was at least 3 months; the participants were postmenopausal women; the effects of soy isoflavone extract or isolated soy protein with isoflavones were tested on lipids in both treatment arms.

The exclusion criteria were as follows: studies with cross-over design; men, men and women or premenopausal women as participants; insufficient quantitative data; study duration of less than 12 weeks; isoflavones mixed with other active formulations; duplicate reports. The search process was independently carried out by two or more investigators; all screening conflicts were resolved by consensus throughout the research team.

### 2.2. Data Extraction

Data were extracted by the lead author and subsequently reviewed by the co-authors for accuracy. Extracted data included: first author’s name, year of publication, country of origin, follow-up period of the study, age (range), menopause status (years since menopause), body mass index; daily dose of soy isoflavones in the active arm (aglycone equivalent; clearly described composition of isoflavones and their doses); type of control group; information concerning the baseline and final of mean concentrations of components of the lipid profile, as well as their standard deviation (SD) or standard error (SE) or 95% confidence intervals (95% CI) and group size (*n*) in each test arm. The analysis included all the multi-arm study intervention groups that were relevant for the systematic review. To avoid duplication of data from the same groups with multiple time points, only endpoints with the longest duration were considered. When a few publications were based on the same study, reports with the largest number participants were selected.

### 2.3. Quality Assessment and Bias Risk of the Trials

The quality of trials was evaluated using the Cochrane Collaboration’s tool. This is a listing of seven items that have a potential biasing influence on the estimates of an interventions effectiveness in randomized studies, and includes: random sequence generation, allocation concealment, blinding of participants and personnel, blinding of outcome assessment), attrition bias (incomplete outcome data), reporting bias (selective reporting) and other sources of bias. The risk of bias in RCTs included in review is assessed as: ‘High risk’ or ‘Unclear’ or ‘Low risk’ [24]. To explain the possible presence of bias in the included publications, their funnel plot symmetry was checked, moreover, Begg’s rank correlation test (Kendall Tau) and Egger’s weighted regression test were applied [25,26].

### 2.4. Statistical Analysis and Meta-Analysis

The outcome measures were difference in mean (net change in mmol/L) of elements of the lipid profile between baseline and the end values for both the intervention and control groups. In some studies, the results were reported in mg/dL. These we converted into mmol /L using standard conversion factors (multiplying mg/dL by 0.02586 for TC, LDL-C, HDL-C and 0.0113 for TAG). Data of the size of the effects of soy isoflavones on individual components of the lipid profile in each arm of the study were presented as number of subjects (*n*) and the mean ± SD of the difference in means (MD) (net change in mmol/L) between final and initial value. None of the studies provided sufficient information to allow us to directly calculate the variance of change between pre- and post-intervention values. The missing SDs of MDs were inputted using the methods described in the Cochrane Handbook [24], as suggested by Follman et al. [27] and assuming a correlation coefficient of 0.5. Weighted mean difference (WMD) was calculated by subtracting the difference in mean between the control and active groups. The random-effects model was applied, and 95% CI and *p* < 0.05 were considered statistically significant [28]. STATISTICA Medical Software StatSoft Poland was used for all statistical analyzes. For heterogeneity evaluation, Cochrane Q and I^2^ statistic were employed. The I^2^ test allowed to assess whether the variance cross studies were correct and not due to a sampling error. The percentage of total variation indicates the degree of heterogeneity; I^2^ values of ≤25% were considered low, >25% as moderate and ≥75% were assessed as high heterogeneity [29]. Multivariate meta-regression was also applied.

### 2.5. Subgroup Analysis

An additional analysis was undertaken in order to detect sources of heterogeneity via the following definitions: age of the participants: <55 vs. ≥55 y; BMI: ≤24.9 kg/m^2^ vs. ≥25 kg/m^2^; post-menopausal status: early (<5 y) vs. late (≥5 y); cholesterol: normal vs. borderline vs. high (TC cut-off points: 5.2 mmol/L, 6.2 mmol); follow-up period: <6 vs. ≥6 months; types intervention: soy protein with isoflavones vs soy isoflavone alone; the total dose of isoflavones (expressed as aglycone equivalents): <80 mg/day ≥80 mg/d. To assess the relationship between the above-mentioned variables, to establish which of them account for the heterogeneity and for determining the possible impact of isoflavones on individual variables, we used multivariate meta-regression analysis [30]. The following variables were used in the multivariate meta-regression analysis: age of the participants, BMI, post-menopausal status, cholesterol, follow-up period, types of intervention and the total dose of isoflavones.

## 3. Results

**Figure 1:**
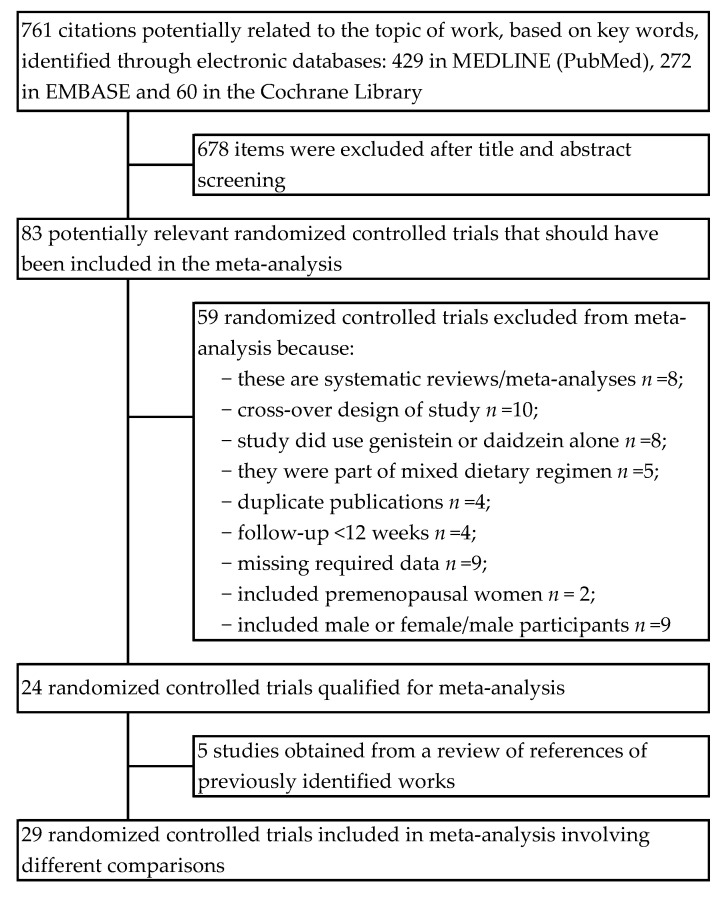
Flowchart of the selection procedure for studies included in the current review and meta-analysis.

In total, 761 citations were identified. Based on the title and/or abstract, 678 items were excluded due to lack of connection with the topic of work. Consequently, 83 potentially relevant clinical trials were qualified for further detailed qualitative analysis in the full-text assessment. Of these, 59 studies were excluded due to the failure to meet all inclusion criteria. As a result, 24 randomized controlled trials were qualified for meta-analysis. These were additionally supplemented with 5 items from the literature review of previously identified articles. Finally, 29 randomized controlled trials with 32 comparisons were included in the meta-analysis [31,32,33,34,35,36,37,38,39,40,41,42,43,44,45,46,47,48,49,50,51,52,53,54,55,56,57,58,59]. Detailed information about of the literature search and study selection and identification can be found in Figure 1.

### 3.1. Assessment of the Methodological Quality of Trials

**Figure 2:**
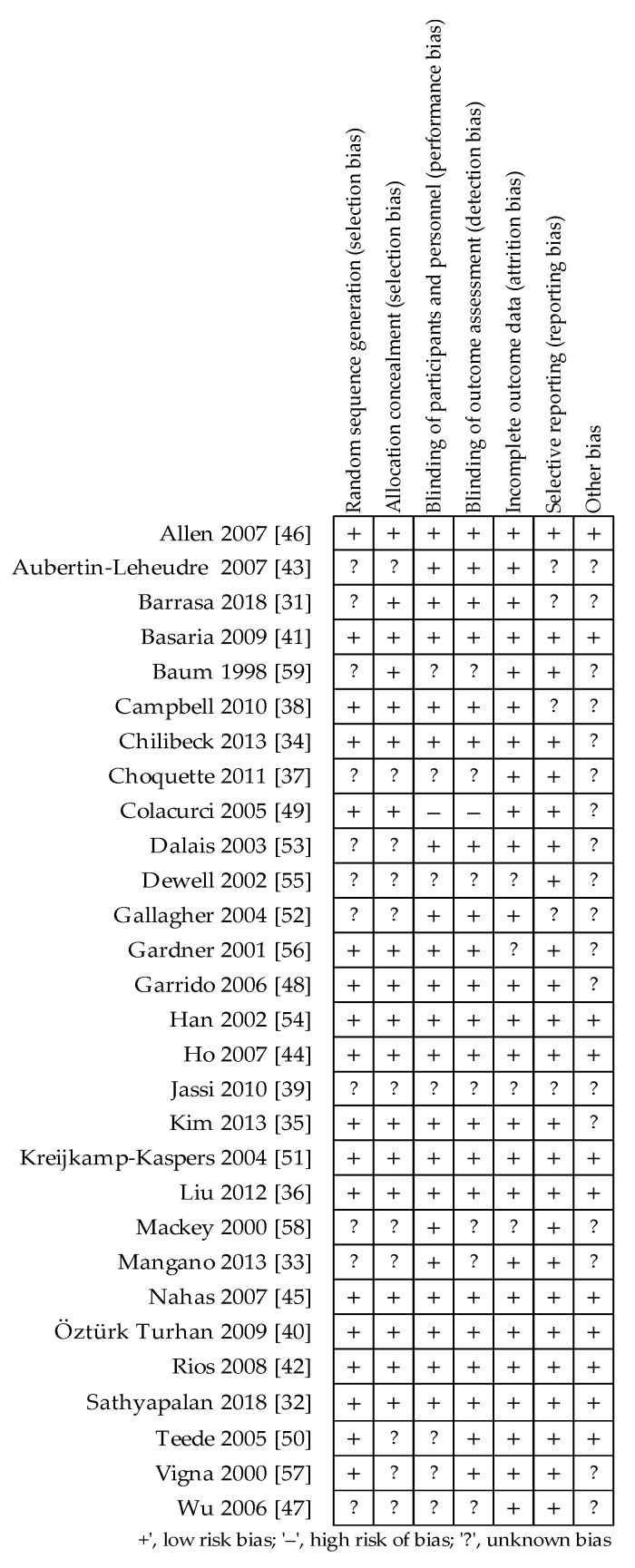
Summary of Cochrane risk of bias for each study [31,32,33,34,35,36,37,38,39,40,41,42,43,44,45,46,47,48,49,50,51,52,53,54,55,56,57,58,59].

**Figure 3:**
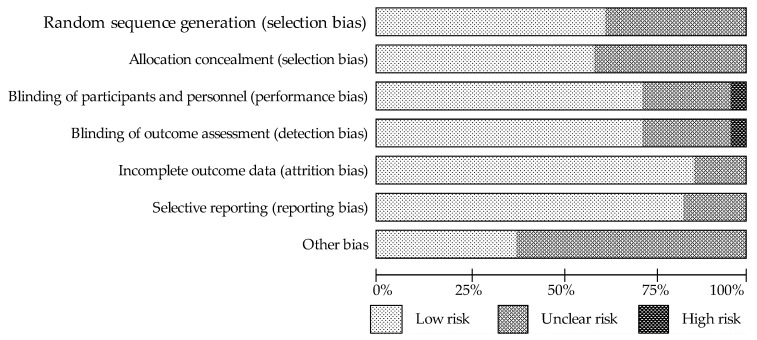
Summary of risk of bias for each item presented as percentages for across all included studies.

The quality of the included studies was evaluated according to the Cochrane Instructions, based on of risk of bias summary for each study (Figure 2) and of risk bias for each item (Figure 3). The studies, to various degrees, described a randomization design and the adapted allocation concealment. With regard to blinding, all studies reported double-blinding and most of them provided a further description of the binding procedure. One of the aforementioned RCTs did not indicate any measures for blinding of outcome assessment [49]. Nevertheless, the vast majority of the evaluated trials showed a low-risk bias for incomplete outcome data and selective outcome reporting.

### 3.2. Characteristics of Included Trials

The characteristics of selected randomized controlled trials analyzing the effects of soy isoflavones on the lipid profile in menopausal women are presented in Table 1. The disclosed analysis included 29 studies published from 1998 to 2018 [31,32,33,34,35,36,37,38,39,40,41,42,43,44,45,46,47,48,49,50,51,52,53,54,55,56,57,58,59]. In three trials, two treatment groups with different doses of isoflavones were compared with one identical control group. These trials were analyzed separately [44,52,58]. One trial involved both male and female participants; the meta-analysis only analyzed the data from the women included in this study [57]. In this case, 11 studies were carried out in North America, five in South America, four in Europe, six in Asia and three in Australia. In 19 including RCTs, the effect of soy isoflavones on lipid was mainly investigated, while in the rest of the trials, research was directly towards the effect of isoflavone supplementation on bone mass [34,47,52], menopausal symptoms relief [45,54], body composition [37], endothelial function [49,50], quality of life and cognition [41,51]. In these, the secondary aim was of our interest—the evaluation of the changes in lipid profiles. Here, 11 articles reported outcomes of studies for durations of 12 weeks, one study was 4 months long, nine were 6 months long, one was 9 months long, five were 12 months long, one spanned 15 months long and one was 24 months in duration.

In total, 2305 postmenopausal women participated in the analyzed studies (1217 in active groups and 1088 in control groups). Mostly, the RCT were conducted among healthy women, while in four studies the women-participants had baseline hypercholesterolemia, according to the definitions of the original study [46,55,56,59] and three trials included participants with various conditions, including overweight/obesity [37,43] and prediabetes [36]. The mean age of the women was 57.2 years (range: 48.5 to 73.9), and mean body mass index was 28 kg/m^2^ (median: 26.2; range: 21.1 to 32.0).

**Table 1:** Characteristics of selected randomized controlled studies assessing the influence of soy isoflavones on lipid profile in postmenopausal women.

First Authors Data [ref.] Country	Study Design Trial Duration	Study Population Health Condition’	*n* Sample (Treated/Control) Placebo	Intervention (Daily Dose)	Dietary Advice During Study:	Group Studied	Baseline Lipids Values			
							TC mmol/L	LDL-C mmol/L	HDL-C mmol/L	TAG mmol/L
Barrasa 2018 [31] Chile	Parallel groups 1-wk run-in / 3-mo follow-up	Mean age 64.7 ± 4.6 (55–72) y, ysm N/A, BMI 27.6 ± 0.9, healthy	20/15	IAE 100 mg (52 mg Gen, 40 mg Dai, 8 mg Gly) vs. placebo	No reported	SG CG	5.13 ± 0.68 4.87 ± 0.62	3.10 ± 0.94 2.97 ± 0.50	1.30 ± 0.43 1.18 ± 0.38	1.53 ± 0.39 1.54 ± 0.36
Sathyapalan 2018 [32] Great Britain	Parallel groups 6-mo follow-up	Mean age 52 (49–56) y, ysm < 2, BMI 25.4, healthy	60/60	SP 15 g, IAE 66 mg (54% Gen, 35% Dai, 12% Gly) vs. SP 15 g	Avoiding other dietary products containing soy	SG CG	5.8 ± 0.9 5.8 ± 0.8	3.65 ± 0.7 3.65 ± 0.9	1.68 ± 0.94 1.78 ± 0.42	1.16 ± 0.62 1.18 ± 0.57
Mangano 2013 [33] USA	Parallel groups 12-mo follow-up	Mean age 73.9 ± 5.9 (>60) y, ysm 23.1 ± 9.0, BMI 28.8 ± 5.8, healthy	25/22	SP 18 g + IC 105 mg (0.61% Agl) vs. control (MP 18 g + placebo)	Avoiding soy foods, nutritional or herbal supplements	SG CG	5.45 ± 0.87 5.46 ± 1.29	3.50 ± 0.77 3.57 ± 1.13	1.39 ± 0.32 1.33 ± 0.33	1.23 ± 0.62 1.23 ± 0.54
Chilibeck 2013 [34] Canada	Parallel groups 24-mo follow-up	Mean age 56.6 ± 6.8 y, ysm N/A, BMI 27.1 ± 4.1, healthy	72/73	IC 165 mg (105 mg Agl: Gen, Dai and Gly in ratio of 1:1:0.2) vs. placebo	No reported	SG CG	5.87 ± 0.96 5.76 ± 0.91	3.68 ± 0.91 3.59 ± 0.89	1.58 ± 0.41 1.52 ± 0.44	1.41 ± 1.03 1.43 ± 0.79
Kim 2013 [35] Republic of Korea	Parallel groups 12-wk follow-up	Mean age 53.6 ± 3.4 y, ysm 3.6 ± 2.4, BMI 23.3 ± 2.5, healthy	42/43	IC 70 mg (Glyco: 38 mg glycitin, 20 mg daidzin, 12 mg genistin) vs. placebo	Limitation of soy products	SG CG	5.13 ± 0.85 5.48 ± 1.03	2.97 ± 0.70 3.25 ± 0.92	1.49 ± 0.36 1.52 ± 0.37	1.26 ± 0.72 1.27 ± 0.66
Liu 2012 [36] Hong Kong SAR	Parallel groups 2-wk run-in / 3- mo follow-up	Mean age 56.3 ± 4.3 (48–70) y, ysm 5.9 ± 5.4, BMI 24.4 ± 3.6, prediabetes	60/60	SP 15 g, IAE 100 mg (59 mg Gen,4 mg Gly, 35 mg Dai) vs. MP 15 g	Other phytoestrogen supplements were prohibited	SG CG	5.83 ± 0.94 5.63 ± 0.93	3.94 ± 0.67 3.81 ± 0.88	1.66 ± 0.31 1.65 ± 0.30	1.35 ± 1.19 1.30 ± 0.70
Choquette 2011 [37] Canada	Parallel groups 6-mo follow-up	Mean age 58.5 ± 5.5 (50–70) y, ysm 9.0 ± 7.0, BMI 30.1 ± 2.7 overweight/obesity	23/22	IAE 70 mg (44 mg Dai, 16 mg Gly,10 mg Gen) vs. placebo	Maintaining normal eating habits	SG CG	5.40 ± 0.88 5.58 ± 0.86	3.34 ± 0.75 3.34 ± 0.81	1.49 ± 0.34 1.57 ± 0.32	1.47 ± 0.67 1.44 ± 0.73
Campbell 2010 [38] USA	Parallel groups 12-mo follow-up	Mean age 54.7 ± 5.5 (<65) ys, ysm 5.5 ± 5.0; BMI 27.9 ± 5.9, hypercholesterolemic	35/27	SP 25 g, 60 mg IF vs. CP 25 g	Maintaining normal eating habits	SG CG	5.97 ± 0,93 6.15 ± 0.91	3.88 ± 0.90 3.95 ± 0.87	1.47 ± 0.38 1.50 ± 0.36	1.34 ± 0.70 1.48 ± 0.67
Jassi 2010 [39] India	Parallel groups 12-wk follow-up	Mean age 51.1 ± 8.6 (40–60) y, ysm 2.3 ± 1.2, BMI 23.4 ± 2.7, healthy	25/25	SP 30 g, IF 60 mg vs. CP 30 g	No reported	SG CG	4.96 ± 0.36 4.69 ± 0.71	3.09 ± 0.37 2.83 ± 0.76	1.06 ± 0.15 1.06 ± 0.16	1.76 ± 0.28 1.76 ± 0.17
Öztürk Turhan 2009 [40] Turkey	Parallel groups 6-mo follow-up	Mean age 51.5 ± 5.1 (44–58) y, ysm 3.6 ± 1.7, BMI 27.1 ± 3.1, healthy	45/45	IAE 40 mg (29.8 mg Gen, 7.8 mg Dai, 2.4 mg Gly) vs. placebo	Not given products with presumed estrogenic activity	SG CG	6.82 ± 0.96 6.30 ± 0.76	4.25 ± 0.73 4.01 ± 0.65	1.54 ± 0.35 1.38 ± 0.28	1.70 ± 0.53 1.78 ± 0.74
Basaria 2009 [41] USA	Parallel groups 12-wk follow-up	Mean age 55.7 ± 1.3 (46–76) y, ysm 5.7 ± 0.9, BMI 26.1 ± 0.8, healthy	38/46	SP 20 g, IC 160 mg (IAE: 64 mg Gen, 63 mg Dai, 34 mg Gly) vs. MP 20 g	Avoiding products: soy, black cohosh, etc.	SG CG	5.48 ± 0.14 5.69 ± 0.85	3.15 ± 0.75 3.21 ± 0.74	1.88 ± 0.46 2.02 ± 0.46	1.03 ± 0.58 0.99 ± 0.46
Rios 2008 [42] Brazil	Parallel groups 6-mo follow-up	Mean age 55.5 ± 5.2 (47–66) y, ysm 8.8 ± 7.5, BMI 26.5 ± 3.3, healthy	25/22	IC 40 mg (5% Gen, 12% Dai) vs. placebo	Exclusion dietary products high in phytoestrogens	SG CG	5.30 ± 0.90 5.77 ± 1.52	3.41 ± 0.81 3.85 ± 1.36	1.28 ± 0.27 1.27 ± 0.22	N/A N/A
Aubertin-Leheudre 2008 [43] Canada	Parallel groups 6-mo follow-up	Mean age 57.4 ± 5.4 (50–70) y, ysm 8.6 ± 7.5, BMI 32.0 ± 12.5, obesity	21/18	IAE 70 mg (44 mg Dai, 16 mg Gly,10 mg Gen) vs. placebo	Maintaining normal eating habits	SG CG	5.41 ± 0.88 5.33 ± 0.83	3.17 ± 0.81 3.17 ± 0.78	1.55 ± 0.49 1.45 ± 0.37	1.51 ± 0.69 1.52 ± 0.69
Ho 2007 [44] China	Parallel groups 2-wk run-in / 12-mo follow-up	Mean age 54.2 ± 3.1 (48–62) y, ysm 4.1 ± 2.4, BMI 24.1 ± 3.6, healthy	67/68/68	a. IAE 80 mg; b. IAE 40 mg (46.4% Dai, 38.8% Gly, 14.7% Gen) vs. placebo	Maintaining normal eating habits	SG80 SG40 CG	5.86 ± 0.83 5.83 ± 0.84 5.93 ± 0.89	3.19 ± 0.74 3.23 ± 0.68 3.25 ± 0.73	1.89 ± 0.41 1.80 ± 0.39 1.86 ± 0.42	1.13 ± 0.56 1.32 ± 0.93 1.29 ± 0.96
Nahas 2007 [45] Brazil	Parallel groups 4-wk run-in / 4-mo follow-up	Mean age 55.7 ± 6.8 (>45) y, ysm 6.9 ± 4.5, BMI 29.1 ± 5.0, healthy	38/38	IC 100 mg (50% Gen, 35% Dai) vs. placebo	A diet rich in fiber, soy or of vegetarian was banned	SG CG	5.56 ± 0.92 5.37 ± 0.97	3.47 ± 0.82 3.26 ± 0.82	1.29 ± 0.27 1.35 ± 0.34	1.73 ± 0.74 1.67 ± 0.89
Allen 2007 [46] USA	Parallel groups 4-wk run-in / 12-wk follow-up	Mean age 56.8 ± 5.6 y, ysm 9.4 ± 8.3, BMI 27.9 ± 4.7 hypercholesterolemia	93/98	SP 20 g, IC 160 mg (~96 mg Agl) vs. MP 20 g	Low-fat diet	SG CG	5.80 ± 0.68 5.71 ± 0.64	3.67 ± 0.68 3.60 ± 0.57	1.56 ± 0,37 1.52 ± 0.31	1.25 ± 0.51 1.28 ± 0.60
Wu 2006 [47] Japan	Parallel groups 6-mo follow-up	Mean age 54.4 ± 2.9 (45–60) y, ysm N/A, BMI 21.1 ± 2.4, healthy	25/29	IC 75 mg (47 mg Agl: Dai 38.3 mg, Gen 8.6 mg, Gly 1 mg) vs. placebo	No changes in dietary habits	SG CG	5.90 ± 0.76 5.88 ± 0.86	3.52 ± 0.72 3.59 ± 0.76	1.92 ± 0.47 1.85 ± 0.38	0.95 ± 0.43 1.16 ± 0.53
Garrido 2006 [48] Chile	Parallel groups 12-wk follow-up	Mean age 53.5 ± 4.0 (45–60) y, ysm N/A, BMI 26.9 ± 2.3, healthy	15/14	IAE ~100 mg (46,8 mg Dai, 48.2 mg Gen) vs. placebo	Herbal supplements or soy products were prohibited	SG CG	5.5 ± 1.0 4.8 ± 0.5	3.4 ± 0.4 2.9 ± 0.3	1.4 ± 0.3 1.8 ± 0.6	1.3 ± 0.2 1.4 ± 0.2
Colacurci 2005 [49] Italy	Parallel groups 6-mo follow-up	Mean age 55.1 ± 3.8 y, ysm 4.9 ± 0.6, BMI 25.9 ± 1.8, healthy	29/28	IAE 60 mg (30 mg Gen, 30 mg Dai) vs. placebo	Other soy products were prohibited	SG CG	N/A N/A	3.7 ± 0.3 3.6 ± 0.4	1.6 ± 0.5 1.5 ± 0.5	1.5 ± 0.6 1.6 ± 0.8
Teede 2005 [50] Australia	Parallel groups 3-d run-in / 3-mo follow-up	Mean age 59.5 ± 4.5 (50–75) y, ysm N/A, BMI 25.9 ± 5.4, healthy	19/21	SP 40 g, IC 118 mg (54 mg Gen, 3.6 mg Gly, 26 mg Dai) vs. CP 40 g	Dietary items high in phytoestrogens were excluded	SG CG	6.2 ± 1.30 5.8 ± 0.92	4.0 ± 0.87 3.6 ± 0,92	1.6 ± 0.43 1.6 ± 0.46	1.0 ± 0.48 1.0 ± 0.63
Kreijkamp-Kaspers 2004 [51] Netherlands	Parallel groups 12-mo follow-up	Mean age 66.6 ± 4.7 (60–75) y, ysm 17.9 ± 6.9, BMI 26.1 ± 3.8, healthy	88/87	SP 25.6 g, IAE 99 mg (52 mg Gen, 6 mg Gly, 41 mg Dai) vs. MP 25,6 mg	After consultation, possible changes in the diet possible changes in the diet	SG CG	6.21 ± 0.73 6.11 ± 0.95	4.16 ± 0.99 4.12 ± 0.88	1.55 ± 0.41 1.53 ± 0.34	1.36 ± 0.72 1.25 ± 0.59
Gallagher 2004 [52] United States	Parallel groups 1 wk run-in / 15-mo follow-up	Mean age 55.4 ± 1.2 (40–62) y, ysm 7.6 ± 1.3, BMI 26.4 ± 9.8, healthy	17/19/14	SP 40 g: a. IC 96 mg (52 mg Gen, 28 mg Dai); b. IC 52 mg (28 mg Gen, 20 mg Dai) vs. SP 40 g	Restricted animal protein	SG96 SG52 CG	5.70 ± 0.88 7.04 ± 0.59 5.49 ± 1.32	3.57 ± 0.81 3.50 ± 0.83 3.48 ± 1.31	1.42 ± 0.33 1.47 ± 0.34 1.44 ± 0.27	1.56 ± 0.91 1.53 ± 0,82 1,24 ± 0.47
Dalais 2003 [53] Australia	Parallel groups 3-mo follow-up	Mean age 60 ± 6.2 (50–75) y ysm N/A, BMI 25.3 ± 4.6, healthy	38/40	SP 40 g, IC 118 mg (69 mg Agl) vs. CP 40 g	No reported	SG CG	6.12 ± 0.92 5.92 ± 0.88	4.00 ± 0.86 3.69 ± 0.88	1.63 ± 0.49 1.72 ± 0.51	1.09 ± 0.68 1.01 ± 0.57
Han 2002 [54] Brazil	Parallel groups 4-mo fallow-up	Mean age 48.5 ± 7.6 (45–55) y, ysm 1.9 ± 1.6, BMI 24.3 ± 3.2, healthy	40/40	SP 50.3 mg, IAE 33.3 mg (23.3 mg Gen, 3.8 mg Gly, 6.2 mg Dai) vs. placebo	No reported	SG CG	5.83 ± 0.88 5.86 ± 1.26	3.45 ± 0.87 3.45 ± 1.32	1.04 ± 0.23 1.03 ± 0.21	2.31 ± 1.66 1.99 ± 1.66
Dewell 2002 [55] USA	Parallel groups 2-mo follow-up	Mean age 69.5 ± 4.2 (64–83) y, ysm N/A, BMI 25.0 ± 4.2 moderate hypercholesterolemia	20/16	IC 150 mg (90 mg Agl: 45% Gen, 55% Dai and Gly) vs. placebo	Diet excluding foods containing soy	SG CG	6.8 ± 0.9 6.3 ± 2.0	5.6 ± 0.9‡ 5.1 ± 2.0‡	1.2 ± 0.5 1.2 ± 0.4	0.8 ± 0.5 1.3 ± 0.8
Gardner 2001 [56] USA	Parallel groups 4-wk run-in / 12-wk follow-up	Mean age 59.9 ± 6.6 (<80) y, ysm N/A, BMI 26.3 ± 4.6, hypercholesterolemia	31/30	SP 42 g, IAE 80 mg (52 mg Gen, 25 mg Dai, 4 mg Gly) vs. MP 42 g	Diet excluding foods containing soy	SG CG	5.9 ± 0.6 6.1 ± 0.6	3.9 ± 0.6 4.0 ± 0.5	1.5 ± 0.3 1.5 ± 0.4	1.3 ± 0.8 1.3 ± 0.7
Vigna 2000 [57] Italy	Parallel groups 12-wk follow-up	Mean age 53.4 ± 3.3, ysm 2.4, BMI 25.9 ± 3.5, healthy	40/37	SP 40 g, IF 76 mg vs. CP 40 g	No reported	SG CG	6.37 ± 1.01 6.55 ± 0.93	4.13 ± 0.87 4.33 ± 0.87	1.57 ± 0.36 1.61 ± 0.38	1.47 ± 0.90 1.32 ± 0.77
Mackey 2000 [58] Australia	Parallel groups 4-wk run-in / 12-wk follow-up	Mean age 56.6 ± 4.6 (45–65) y, ysm N/A, BMI N/A hypercholesterolemia	25 /24	SP 28 g, IF 65 mg vs. SP 28 g	Dietary guidelines from National Heart Foundation	SG CG	7.29 ± 0.90 7.47 ± 1.04	5.07 ± 0.73 5.11 ± 1.02	1.52 ± 0.39 1.66 ± 0.45	1.53 ± 0.82 1.54 ± 0.77
Baum 1998 [59] USA	Parallel groups 2-wk run-in / 12-wk follow-up	Mean age 60.8 ± 8.6 (49–83) y, ysm N/A, BMI 27.8 ± 5.3, hypercholesterolemia	21/23/22	SP 40 g: a. IAE 90 mg; b. IAE 56 mg vs. CP + MP 40 g	Low-fat diet	SG90 SG56 CG	6.47 ± 0.88 6.57 ± 0.85 6.26 ± 0.67	5.1 ± 1.0‡ 5.2 ± 0.9‡ 4.9 ± 0.8‡	1.38 ± 0.32 1.34 ± 0.28 1.38 ± 0.31	1.74 ± 0.75 1.89 ± 1.02 1.75 ± 1.11

Abbreviations: Agl, aglycones; BMI, body mass index (kg/m^2^); CG, control group: CP, casein protein; Dai, daidzein; FSH, follicle-stimulating hormone; Gen, genistein; Gly, glycitein; Glyc, glycoside; HDL-C, high-density lipoprotein cholesterol; IAE, isoflavone aglycone equivalents; IC, isoflavone conjugate containing aglycone and glycoside; IF, isoflavones (form and composition unknown); MP, milk protein; N/A, not available, NCEP SI/II, National Cholesterol Education Program Step I/II; ref., reference; SG, soy group; SP, soy protein; y, year or years; ysm, years since menopause.

### 3.3. Interventions

Different types of interventions were conducted. In 14 trials, soy isoflavones extract in the form of tablets or capsules was administered and compared with placebo [31,34,35,37,40,42,43,44,45,47,48,49,54,55], while 14 trials used isolated soy protein containing isoflavones [32,36,38,39,41,46,50,51,52,53,56,57,58,59] and were compared with a control group that was either: casein [39,46,50,53,57,59], milk protein [36,41,51,56] or isolated soy protein with or without trace isoflavone content [32,52,58]. One study dealt with interventions that were a combination of soy protein of powder and soy isoflavones tablets—this was compared with a mix of proteins and maltodextrin tablets as controls [33]. The protein in these studies was in powder form and was mixed by participants with water or beverages and/or added to the usual diet or taken in in the form of a snack. Overall isoflavone concentrations averaged 87.6 mg/d (median: 80 mg; range: 30.3 to 165 mg). The range of soy protein was 15 to 50.3 g/d; median: 40 g/d.

### 3.4. Meta-Analysis

**Figure 4:**
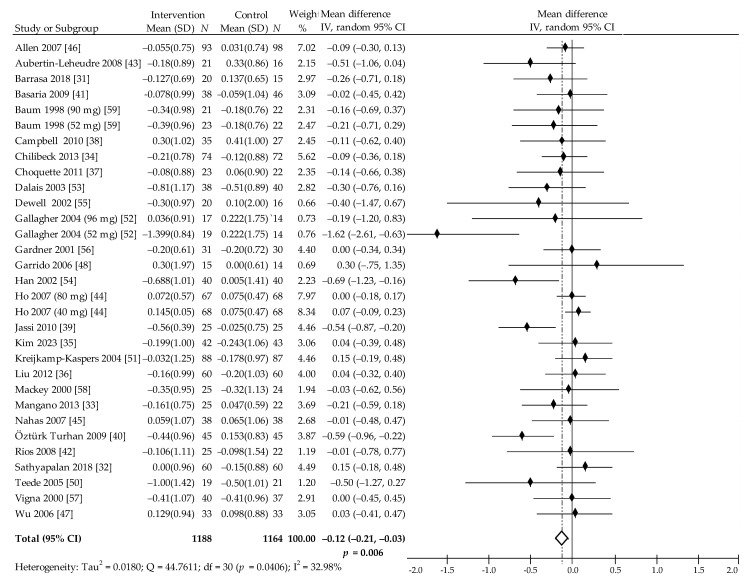
Forest plot showing the effects of soy isoflavones compared with placebo on TC concentrations (mmol/L; change from baseline). Data are presented as weighted mean difference and 95% CI [31,32,33,34,35,36,37,38,39,40,41,42,43,44,45,46,47,48,50,51,52,53,54,55,56,57,58,59].

Overall, our meta-analysis looked at 29 trials with 32 comparisons assessing the influence of isolated soy protein containing isoflavones and/or of soy isoflavones extract on individual components of lipid profiles. However, 28 trials with 31 comparisons provided data for the meta-analysis of impact on TC [31,32,33,34,35,36,37,38,39,40,41,42,43,44,45,46,47,48,50,51,52,53,54,55,56,57,58,59]. One study by Colacurci et al. [49] did not have the required data to be included in the meta-analysis. Our work shows that the value of TC decreased in the isoflavone intake group as compared with the corresponding control group in 17 from 31 comparisons, but only in 4 was a statistically significant reduction evident [39,40,52,54]. In contrast, 3 comparisons showed no change and 8 indicated an insignificant increase. The pooled estimate reveals that the intake of soy protein and/or isoflavones is associated with a statistically significant decrease in TC by −0.12 (95% CI: −0.21 to −0.03) mmol/L, −4.64 (95% CI: −8.12 to −1.16) mg/dL, *p* = 0.007, Q = 44.76, I^2^ = 32.98% (Figure 4). Here, the Begg and Mazumdar’s test for rank correlation was Kendall’s tau = −0.3462, z = −2.7364, *p* = 0.006, indicating possible publication bias, while in Egger’s test for a regression, intercept = −11644 (95% CI = −20814 to −02474), *p* = 0.024, also indicating possible publication bias.

In the subgroup analysis, reduction of TC was significant when follow-up was less than 6 months (*p* = 0.006), in late postmenopausal women (*p* = 0.026), in women older than 55 years (*p* = 0.037), in subjects that were overweight/obese (*p* = 0.012) and when taking soy protein with isoflavones (*p* = 0.024) and isoflavones at a dose <80 mg per day (*p* = 0.024) (Table 2). Multivariate meta regression with all covariates had no significant impact on TC.

**Figure 5:**
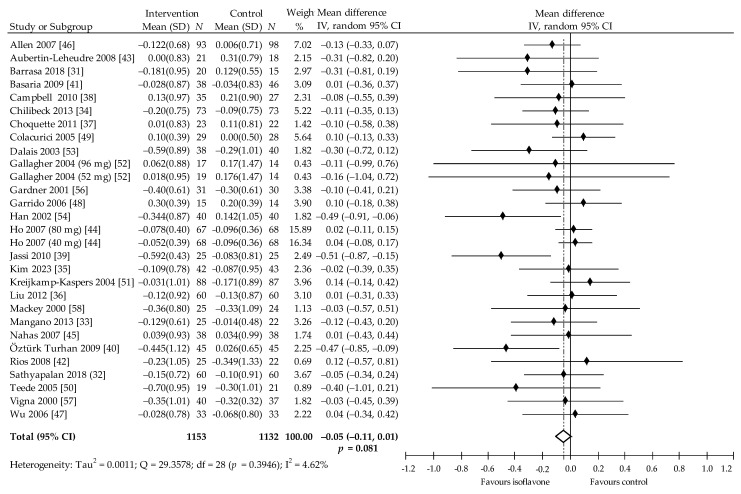
Forest plot showing the effects of soy isoflavones compared with placebo on LDL-C concentrations (mmol/L; change from baseline). Data are presented as weighted mean difference and 95% CI [31,32,33,34,35,36,37,38,39,40,41,42,43,44,45,46,47,48,49,50,51,52,53,54,56,57,58].

In turn, 38 comparisons from 24 trials focused on assessing the effect of soy isoflavones on LDL-C [31,32,33,34,35,36,37,38,39,40,41,42,43,44,45,46,47,48,49,50,51,52,53,54,56,57,58]. Two studies by Dewell et al. [55] and Baum et al. [59] were excluded from the meta-analysis because they lacked adequate data or the data was presented as non-HDL. In the included studies, 16 comparisons, compared to control, showed insignificant decrease in LDL-C concentration as a result of isoflavone consumption, while statistically significant reduction was indicated in three [39,40,54], and in 10 comparisons, insignificant increase was noted. The pooled estimate reveals that the intake of soy protein and/or soy isoflavones is associated with insignificant decrease in LDL-C by −0.05 (95% CI: −0.11 to 0.01) mmol/L, −1.93 (95% CI: −4.25 to 0.39) mg/dL, *p* = 0.081, Q = 29.36, I^2^ = 4.62%. (Figure 5). Here, Begg and Mazumdar’s test for rank correlation had Kendall’s tau = −0.2512, z = −1.9133, *p* = 0.056, indicating no evidence of publication bias. However, Egger’s test for a regression intercept was −0.9868 (95% CI = −1.7445 to −0.2292), *p* = 0.013, indicating possible publication bias.

In the subgroup analysis, decrease of LDL-C was significant when follow-up was less than 6 months (*p* = 0.012), in women older than 55 years (*p* = 0.035) and when taking soy protein with isoflavones (*p* = 0.011), (Table 2). Multivariate meta regression with most covariates showed that these had no significant impact, but age <55 years was found to have a significant influence on LDL-C (*p* = 0.045).

**Figure 6:**
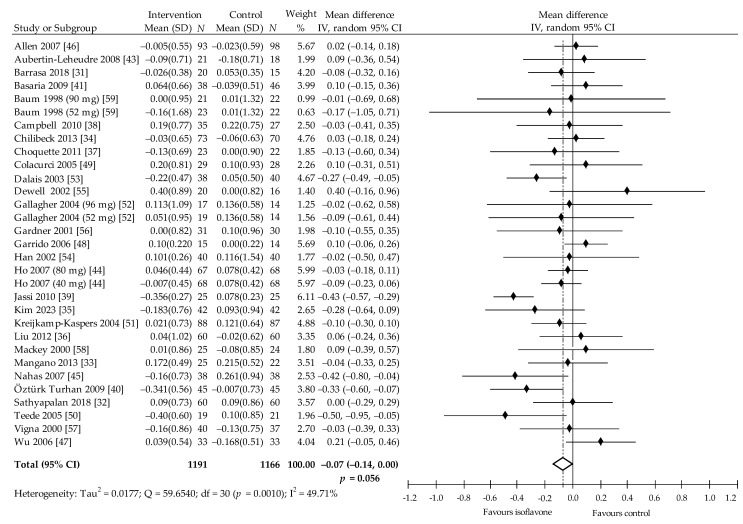
Forest plot showing the effects of soy isoflavones compared with placebo on TAG concentrations (mmol/L; change from baseline). Data are presented as weighted mean difference and 95% CI [31,32,33,34,35,36,37,38,39,40,41,43,44,45,46,47,48,49,50,51,52,53,54,55,56,57,58,59].

Changes in level of TAG after soy isoflavone intake as compared with control group was evaluated in 28 trials (31 comparisons) [31,32,33,34,35,36,37,38,39,40,41,43,44,45,46,47,48,49,50,51,52,53,54,55,56,57,58,59]. One study by Rios et al. [42] did not have the required data to be included in the meta-analysis. In 15 comparisons, compared with control, TAG demonstrated insignificant reduction; in 5, a statistically significant decrease was evident [39,40,45,50,53]; in one study, no changes were observed [32]; in 10, an insignificant increase in TAG levels was noted. The pooled estimate reveals that intake of soy protein and/or isoflavones is associated with a decrease in TAG (with marginal statistical significance): −0.07 (95% CI: −0.14 to 0.00) mmol/L, −6.123 (95% CI: −12.25 to 0.00) mg/dL, *p* = 0.056, Q = 59,65, I^2^ = 49.71% (Figure 6). The Begg and Mazumdar’s test for rank correlation had Kendall’s tau = −0.0538, z = −0.4249, *p* = 0.671, indicating no evidence of publication bias. Egger’s test for regression intercept was 0.394 (95% CI −0.7938 to 15,829), *p* = 0.502 also indicating no evidence of publication bias.

The results of subgroups analysis did not demonstrate any statistically significant differences (Table 2). Multivariate meta regression with all covariates showed no significant impact on TAG, albeit, close to statistical significance was noted for TAG (*p* = 0.052) when taking isoflavones at a dose ≥80 mg/dl.

**Figure 7:**
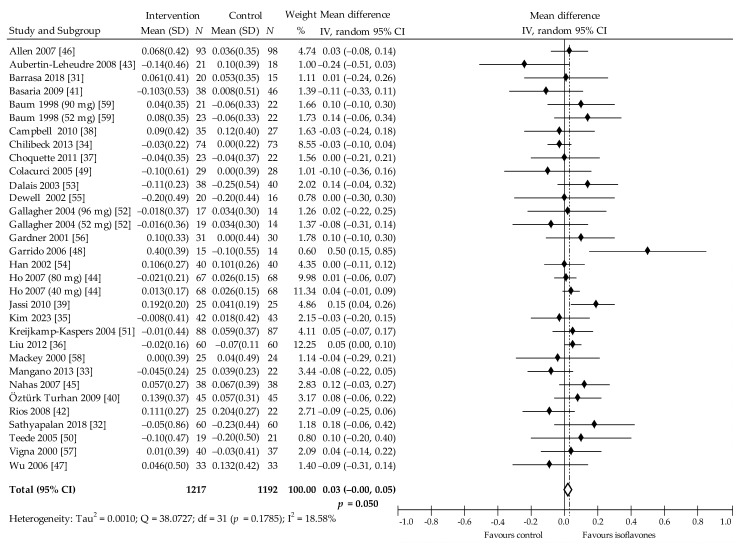
Forest plot showing the effects of soy isoflavones compared with placebo on HDL-C concentrations (mmol/L; change from baseline). Data are presented as weighted mean difference and 95% CI [31,32,33,34,35,36,37,38,39,40,41,42,43,44,45,46,47,48,49,50,51,52,53,54,55,56,57,58,59].

The change in concentrations of HDL-C post-intervention was based on 29 trials (32 of which were comparisons) [31,32,33,34,35,36,37,38,39,40,41,42,43,44,45,46,47,48,49,50,51,52,53,54,55,56,57,58,59]. In 15 comparisons, the comparison with controls demonstrated insignificant increase in HDL-C, while three revealed statistically significant increase [36,39,48]. Moreover, in three studies, no changes were observed [37,54,55], and in 11 studies, insignificant decrease in HDL-C levels was evident. The pooled estimate indicated that the intake of soy protein and/or isoflavones is associated with increase in HDL-C at 0.03 (95% CI: 0.00 to 0.05) mmol/L, 1.15 (95% CI: 0.00 to 1.93) mg/dL, *p* = 0.050, Q = 38.07, I^2^ = 18.58% (Figure 7). Begg and Mazumdar’s test for rank correlation had Kendall’s tau = −0.0565, z = −0.4541 *p* = 0.650, indicating no evidence of publication bias. Egger’s test for regression intercept was −0.0401 (95% CI −0.8003 to 0.7202), *p* = 0.915, also indicating no evidence of publication bias.

In the subgroup analysis, increase of HDL-C was significant when follow-up was less than 6 months (*p* = 0.001), in women overweight/obese (*p* = 0.019) and when taking soy protein containing isoflavones (*p* = 0.003) (Table 2). Furthermore, multivariate meta-regression showed a close to statistical significance effect on HDL (*p* = 0.055) in women with normal body weight.

## 4. Discussion

The present meta-analysis demonstrates that the intake by postmenopausal women of soy protein containing isoflavone and soy isoflavone extract is associated with a significant decrease in serum TC (−0.12 mmol/L, *p* = 0.01), increase of HDL-C (0.03 mmol/L, *p* = 0.05), albeit linked with insignificantly reduction in LDL-C (−0.05 mmol/L, *p* = 0.081) and TAG (−0.07 mmol/L, *p* = 0.06). These findings are generally consistent with previous published meta-analyses for the effect on serum lipid components. These revealed that soy protein and /or isoflavones (compared with control) were more effective in generating changes of lipid profile in older women. In the meta-analysis by Zhan and Ho [60], the effect of soy protein containing isoflavones supplementation on serum lipid level in postmenopausal women (in a subgroup according to gender) was: −0.13 mmol/L, *p* = 0.06 for TC, −0.15 mmol/L, *p* = 0.03 for LDL-C, 0.05 mmol/L, *p* = 0.04 for HDL-C and −0.07 mmol/L, *p* = 0.04 for TAG. In turn, in their meta-analysis, Prediger et al. [61] reported that used of soy protein with isoflavones in women (mostly postmenopausal) was associated with a significant decrease in TC (−0.14 mmol/L, *p* = 0.035), and no significant associations for LDL-C (−0.09 mmol/L, *p* = 0.155), TAG (−0.09 mmol/L, *p* = 0.09) and HDL-C (0.023 mmol/L, *p* = 0.44). The outcomes of other meta-analysis investigating the effects of soy-associated isoflavones on serum lipids in both men and women remain inconsistent and controversial [60,62,63,64,65,66,67]. Yeung and Yu [62], for example, found no overall statistical and clinical benefit from taking soy-associated isoflavones. The aforementioned results were confirmed by Weggemans, Trautwein [63] and Sacks et al. [16]. In contrast, Taku et al. [64] reported positive effect of supplementation of isoflavones on individual lipid components. Similar results were observed by Tokede et al. [65] and Reynolds et al. [66]. Simental-Mendía et al. [67] noted a significant reduction in TC and LDL-C concentrations, whereas levels of HDL-C and TAG remained unaffected.

The strongest lowering impact on TC, LDL-C and TAG, as well as increases in HDL-C values were observed when soy protein with isoflavones was administered. Other authors observed a similar effect [60,65]. There were no significant changes in lipids in subjects taking tablets/capsules containing extracted soy isoflavones, which was also confirmed in the analysis of Zhan and Ho [60]. One possible explanation for the absence of clear impact of soy isoflavones extract on lipid concentrations may be associated with the use of preparations with differences in composition and content of soy isoflavones, especially in the form of the aglycons: daidzein, genistein and glycitein [68]. Variations in their bioavailability should, therefore, be taken into account as one may be more effective than the others in affecting the components of the lipid profile [69]. In addition, it is possible, that several other soy compounds have effect on lipid level. Among these are the proteins and associated trypsin inhibitors, phytic acid and saponins, however, their exact action is not well elicited yet [70].

Our results of subgroup analysis indicate that significant lowering effects of soy preparations on individual components of the lipid profile occur within the shorter follow-up period, compared with durations of more than 6 months. The observed difference between changes in the observation period is unclear. This may be associated with a decrease in compliance with dietary discipline in the extended research period. Similar observations were noted in other works [60,65]. A subgroup analysis of women in the period of late postmenopause and over the age 55 years showed a clear lowering in the level of TC and LDL-C, suggesting that these women may have greater benefits from taking soy preparations. However, initial TC in the participants did not show any major impact on changes in the concentration of the serum lipids.

When considering this meta-analysis, some limitations should be taken into account that may affect its final outcome. First of all, it involved a limited number of subjects, and the small sample size in some studies might have resulted in insufficient statistical power, thus limiting definitive conclusions. Secondly, factors as race, genetic background, environment and lifestyle may also impact on lipid levels after soy therapy. Thirdly, the selected studies used different forms and doses of soy isoflavones and this could affect the final results. Fourthly, the abundance of isoflavones in soy protein preparations varies widely and depends on the processing techniques used during production. Furthermore, the intensity of action of isoflavones may be partly due to the process in which they were extracted [71]. Fifthly, the variability of result of lipid-lowering effect by soy isoflavones may be caused, at least in part by differential equal production among subjects. Finally, the analyzed works might not have represented all the studies related to this subject, especially those published in languages other than English. Hence, it is possible that a study with statistically significant results might have prevailed over a study with an insignificant or zero result, and vice versa. If the results of the published studies are considerably erroneous, the effect of RCI on lipid metabolism might be overestimated or underestimated.

## 5. Conclusions

In conclusion, our systematic review and meta-analysis clearly show that soy isoflavones significantly contribute to beneficial correction of lipid profile in postmenopausal women. Results suggests that soy and its isoflavones can be effective in correction changes in lipid metabolism and may favorably influence in preventing cardiovascular events in postmenopausal women. However, further multicenter studies based on greater amounts of research material and accurately defined doses of isoflavones are necessary to determine their beneficial effect on lipid metabolism, i.e., the lowering of risk of cardiovascular disease in women during this period of life.

## Figures and Tables

**Table 2:** Pooled estimates of treatment effect on lipid profile in subgroups of trials ^a^.

Subgroup Outcome	TC (mmol/L)					LDL-C (mmol/L)					HDL-C (mmol/L)					TAG (mmol/L)				
	*n*	*N*	WMD (95% CI)	*p*	I^2^ (%)	*n*	*N*	WMD (95% CI)	*p*	I^2^ (%)	*n*	*N*	WMD (95% CI)	*p*	I^2^ (%)	*n*	*N*	WMD (95% CI)	*p*	I^2^ (%)
Overall effects	31	2351	−0.12 (−0.21, −0.03)	0.007	32.98	29	2284	−0.05 (−0.01, 0.01)	0.081	4.62	32	2409	0.03 (−0.00, 0.05)	0.050	18.58	31	2397	−0.07 (−0.14, 0.00)	0.056	49.71
**Follow-Up Period**																				
<6 months	16	1143	−0.15 (−0.25, −0.04)	0.006	3.42	14	1055	−0.13 (−0.22, −0.03)	0.012	5.32	16	1143	0.06 (0.03, 0.10)	0.001	9.06	16	1142	−0.12 (−0.24, 0.01)	0.062	65.03
≥6 months	15	1208	−0.11 (−0.25, 0.03)	0.125	48.54	15	1229	−0.01 (−0.07, 0.06)	0.867	0.00	16	1265	0.00 (−0.03, 0.03)	0.899	0.00	15	1215	−0.04 (−0.10, 0.03)	0.299	0.00
Coefficients β (SE), *p* ^b^	<6 mths: −0.097 (0.13), 0.469					≥6 mths: 0.131 (0.09), 0.141					<6 mths: 0.037(0.03), 0.213					<6 mths: −0.129 (0.08), 0.091				
**Postmenopausal Status**																				
<5 years	12	952	−0.13 (−0.30, 0.03)	0.115	59.62	7	672	−0.07 (−0.18, 0.04)	0.192	44.65	13	1009	0.04 (−0.00, 0.09)	0.077	30.11	13	1008	−0.07 (−0.20, 0.05)	0.235	70.35
≥5 years	19	1399	−0.11 (−0.20, −0.11)	0.026	0.00	21	1563	−0.08 (−0.17, 0.01)	0.069	0.00	19	1400	0.02 (−0.02, 0.05)	0.328	11.65	18	1349	−0.06 (−0.13, 0.02)	0.138	5.35
Coefficients β (SE), *p* ^b^	≥5 yrs.: −0.089 (0.15), 0.560					≥5 yrs.: 0.124 (0.11), 0.254					≥5 yrs.: −0.031 (0.04), 0.419					≥5 yrs.: −0.039 (0.10), 0.690				
**Age of Participants**																				
<55 years	16	1201	−0.14 (−0.30, 0.02)	0.079	58.47	17	1258	−0.04 (−0.13, 0.04)	0.338	23.02	17	1258	0.03 (−0.01, 0.07)	0.175	32.64	16	1210	−0.08 (−0.20, 0.03)	0.163	67.02
≥55 years	15	1150	−0.11 (−0.21, −0.01)	0.037	0.00	12	1026	−0.10 (−0.20, −0.01)	0.035	0.00	15	1151	0.03 (−0.01, 0.06)	0.130	1.91	15	1147	−0.05 (−0.13, 0.02)	0.152	0.00
Coefficients β (SE), *p* ^b^	≥55 yrs.: −0.061 (0.13), 0.637					≥55 yrs.: 0.183 (0.91), 0.045					≥55 yrs.: −0.050 (0.03), 0.127					≥55 yrs.: −0.037 (0.08), 0.641				
**Body Mass Index**																				
≤24.9 kg/m^2^	8	708	−0.11 (−0.30, 0.07)	0.226	58.87	7	672	−0.07 (−0.22, 0.07)	0.302	54.63	8	708	0.04 (0.01, 0.07)	0.019	7.75	8	707	−0.06 (−0.24, 0.12)	0.518	78.82
≥25.0 kg/m^2^	22	1594	−0.13 (−0.24, −0.03)	0.012	20.31	21	1563	−0.07 (−0.15, 0.00)	0.060	0.00	23	1652	0.02 (−0.02, 0.07)	0.274	25.13	22	1601	−0.06 (−0.12, 0.01)	0.083	10.02
Coefficients β (SE), *p* ^b^	≤24.9 kg/m^2^: −0.038 (0.13), 0.770					≤24.9 kg/m^2^: −0.051 (0.09), 0.573					≤24.9 kg/m^2^: −0.056 (0.03), 0.055					≤24.9 kg/m^2^: 0.048 (0.07), 0.511				
**Cholesterol**																				
Normal	4	199	−0.23 (−0.56, 0.10)	0.174	46.05	4	199	−0.17 9–0.46, 0.13)	0.270	60.60	4	199	0.12 (−0.04, 0.29)	0.151	63.59	4	198	−0.17 (−0.46, 0.13)	0.263	88.20
Bordeline	18	1564	−0.04 (−0.12, 0.04)	0.304	0.00	18	18	−0.04 (−0.10, 0.02)	0.221	0.00	18	1565	0.01 (−0.02,0.04)	0.339	16.48	17	1514	−0.03 (−0.09, 0.03)	0.294	0.00
High	9	588	−0.27 (−0.53, 0.00)	0.052	56.06	6	464	−0.12 (−0.36, 0.11)	0.289	34.19	9	588	0.05 (−0.01, 0.11)	0.098	0.00	9	588	−0.13 (−0.27, 0.02)	0.090	16.34
Coefficients β (SE), *p* ^b^	Normal: −0.130 (0.19), 0.505					Borderline: −0.095 (0.12), 0.447					Normal: 0.084 (0.05), 0.061					Normal: −0.042 (0.10), 0.685				
	High: −0.219 (0.13), 0.085					High: −0.057 (0.15), 0.700					High: −0.063 (0.034), 0.065					High: −0.001 (0.08), 0.985				
**Types of Intervention**																				
Soy protein ^c^	18	1386	−0.15 (−0.28, −0.02)	0.024	39.41	16	1298	−0.12 (−0.21, −0.03)	0.011	0.00	18	1386	0.05 (0.02, 0.08)	0.003	0.00	18	1386	−0.10 (−0.20, 0.01)	0.066	49.25
Isoflavone extract	13	965	−0.08 (−0.20, 0.03)	0.163	22.82	13	986	−0.00 (−0.07, 0.07)	0.925	0.00	14	1023	0.01 (−0.04, 0.05)	0.768	34.15	32	971	−0.04 (−0.13, 0.06)	0.445	42.18
Coefficients β (SE), *p* ^b^	Isoflavones alone: 0.130 (0.13), 0.301					Dietary isoflavones: −0.139 (0.09), 0.104					Isoflavone alone: −0.019 (0.03), 0.540					Isoflavone alone: −0.069 (0.07), 0.353				
**Isoflavone Dose**																				
<80 mg/day	15	1024	−0.21 (−0.39, −0.03)	0.021	61.91	15	1036	−0.10 (−0.22, 0.01)	0.080	52.44	16	1081	0.02 (−0.02, 0.07)	0.327	1947	15	1033	−0.09 (−0.22, 0.03)	0.149	56.75
≥80 mg/day	16	1327	−0.06 (−0.15, 0.03)	0.185	0.00	14	1248	−0.04 (−0.11, 0.03)	0.294	0.00	16	1328	0.03 (−0.01, 0.07)	0.111	22.84	16	1324	−0.04 (−0.12, 0.04)	0.292	26.99
Coefficients β (SE), *p* ^b^	≥80 mg/d: 0.194 (0.12), 0.115					<88 mg/d: −0.117 (0.08), 0.164					≥80 mg/d: −0.025 (0.03), 0.383					≥80 mg/d: −0.147 (0.08), 0.052				

^a^ Differences in the number of comparisons and sample sizes in some subgroups are due to the lack data in regarding their studies; ^b^ for meta-regression analysis; Abbreviations: CI, confidence interval; I^2^, coefficient of inconsistency; *β*, standardized regression coefficient; *n*, number of comparisons; *N*, number of subjects; *p*, probability value, WMD, weighted mean difference; mths, months; yrs, years.

## Data Availability

Not applicable.

## Supplementary Materials

The following are available online at https://www.mdpi.com/article/10.3390/nu13082531/s1, Table S1: PRISMA 2020 Checklist.
